# Molecular mechanism of the *Escherichia coli* AhpC in the function of a chaperone under heat-shock conditions

**DOI:** 10.1038/s41598-018-32527-7

**Published:** 2018-09-20

**Authors:** Neelagandan Kamariah, Birgit Eisenhaber, Frank Eisenhaber, Gerhard Grüber

**Affiliations:** 1 Bioinformatics Institute, Agency for Science, Technology and Research (A*STAR), 30 Biopolis Street, #07-01 Matrix, Singapore, 138671 Republic of Singapore; 2 School of Computer Engineering, Nanyang Technological University (NTU), 50 Nanyang Drive, Singapore, 637553 Republic of Singapore; 3 School of Biological Sciences, Nanyang Technological University, 60 Nanyang Drive, Singapore, 637551 Republic of Singapore

## Abstract

Peroxiredoxins (Prxs) are ubiquitous antioxidants utilizing a reactive cysteine for peroxide reduction and acting as a molecular chaperone under various stress conditions. Besides other stimulating factors, oxidative- and heat stress conditions trigger their ATP-independent chaperoning function. So far, many studies were intended to reveal the chaperoning mechanisms of the so-called sensitive Prxs of eukaryotes, which are susceptible to inactivation by over-oxidation of its reactive cysteine during H_2_O_2_ reduction. In contrast, the chaperone mechanisms of bacterial Prxs, which are mostly robust against inactivation by over-oxidation, are not well understood. Herein, comprehensive biochemical and biophysical studies demonstrate that the *Escherichia coli* alkyl hydroperoxide reductase subunit C (*Ec*AhpC) acquires chaperone activity under heat stress. Interestingly, their chaperoning activity is independent of its redox-states but is regulated in a temperature-dependent manner. Data are presented, showing that oxidized *Ec*AhpC, which forms dimers at 25 °C, self-assembled into high molecular weight (HMW) oligomers at higher temperatures and supressed aggregation of client proteins at heat-shock conditions. In addition, we unravelled the essential role of the C-terminal tail of *Ec*AhpC on heat-induced HMW oligomer formation and chaperoning activity. Our findings suggest a novel molecular mechanism for bacterial Prxs to function as chaperone at heat-shock conditions.

## Introduction

Microbes encounter a myriad of stresses in their natural environments, including, for pathogens, their hosts. Diverse pathogens have developed exquisite stress response mechanisms that enable their survival and propagation in host systems under stress conditions^1,2^. Reactive oxygen species (ROS) are inevitable by-products produced in the cell through a wide range of physiological processes or when an organism is exposed to a variety of stress conditions^3,4^. Peroxiredoxins (Prxs), a class of thiol-based peroxidases, are a crucial component of the antioxidant defense against ROS. Prxs are most abundant and ubiquitous peroxidases that consist of six evolutionary related subfamilies^5^. They confer major advantages to pathogens through catalysing the rapid reduction of a broad range of peroxide substrates like H_2_O_2_, organic hydroperoxide and peroxynitrite^5,6^.

The Prxs subfamily enzyme AhpC/Prx1, also called 2-Cys Prx, rely on two conserved cysteine residues for the reduction of H_2_O_2_^4^. A highly conserved nucleophilic cysteine located at the N-terminus, also known as peroxidatic cysteine (C_P_), becomes selectively oxidized by peroxide to the C_P_–SOH intermediate and further reacts with a so-called resolving cysteine (C_R_) located at the C-terminus of the other subunit of the AhpC homodimer, forming an intermolecular disulphide^5,7^. AhpC/Prx1 enzymes are obligate homodimers (α_2_), but they are able to modulate the quaternary structure depending on their redox-state. The reduced enzymes have a tendency to form a doughnut shaped ((α_2_)_5_) decamer^8^ or dodecamer^9^, while oxidation weakens the decamer or dodecamer to form dimers^10^. Regeneration of the intermolecular disulphide bond occurs via AhpF, a dedicated AhpC reductase in most bacteria, or thioredoxin, enabling a continuous peroxidase cycle^5,11^. In addition to their antioxidant function, AhpC/Prx1 are anticipated to be involved in a broad range of cellular functions including H_2_O_2_ signaling, protein oxidation and chaperoning function^12–14^.

The chaperone activity of the yeast cytosolic Prx was reported by Jang *et al*.^15^ first, underlining the relationship between the role of oxidative stress and heat shock exposure on its chaperone function. Later, many studies explored other stimuli that trigger the chaperone function of Prxs from various organisms^16–21^. These studies indicated that the C_P_ over-oxidation to C_P_-SO_2_H/SO_3_H under oxidative stress would be one of the several mechanisms that trigger chaperone function. In addition, over-oxidation of Prxs is shown to be essential for the recruitment of Hsp70 chaperones and Hsp104 chaperones to misfolded proteins under oxidative stress, suggesting the crucial role of Prxs in lifespan extension^22^. However, under oxidative stress, Prxs undergo structural rearrangement that enables formation of high molecular weight (HMW) oligomers through stacking of decamers (((α_2_)_5_)_n_), and to exhibit the chaperone activity^18,21^. Interestingly, the studies on mitochondrial Prx of *Leishmania infantum* showed that heat-stress mediated restructuring of the reduced decamers was alone sufficient for its chaperone activity that plays a crucial role in parasite infectivity^23^. Taken together, all these studies indicate that the redox-state of C_P,_ reduced (C_P_-SH) or over-oxidized (C_P_-SO_2_H/SO_3_H), and their associated oligomeric forms, decamer ((α_2_)_5_) or HMW (((α_2_)_5_)_n_), respectively, are crucial for guiding the chaperone activity of Prxs.

In contrast to their sensitive eukaryotic counterparts, most of the bacterial Prxs are more resistant to high H_2_O_2_ concentrations, lacking the controlled over-oxidation mechanism^7,24,25^. In comparison with the large number of studies deciphering the biochemical properties of robust bacterial Prxs under oxidative stress, little is known about their functional role in other stress conditions. In order to explore the role of bacterial Prxs under heat-stress condition, the biochemically well-established *E. coli* AhpC and its mutants were studied to understand their ATP-independent chaperone activity. Using key enzymes for antioxidants or metabolism like the catalase, citrate synthase (CS), and lactate dehydrogenase (LDH) as clients, the studies reveal that *E. coli* AhpC acquires chaperone function, protecting the proteins against heat-induced aggregation and inactivation. Interestingly, we show that the chaperone activity of *Ec*AhpC is temperature dependent, and not dependent on its redox-state. At elevated temperatures, both the oxidized and reduced forms of WT *Ec*AhpC self-assemble into HMW oligomers that essentially supress the thermal aggregation of proteins. In addition, we revealed the essential role of the C-terminal tail of *Ec*AhpC on the heat-induced structural rearrangement to attain its chaperone activity.

## Results

### Oxidized *Ec*AhpC functions as a chaperone under heat shock

**Figure 1:**
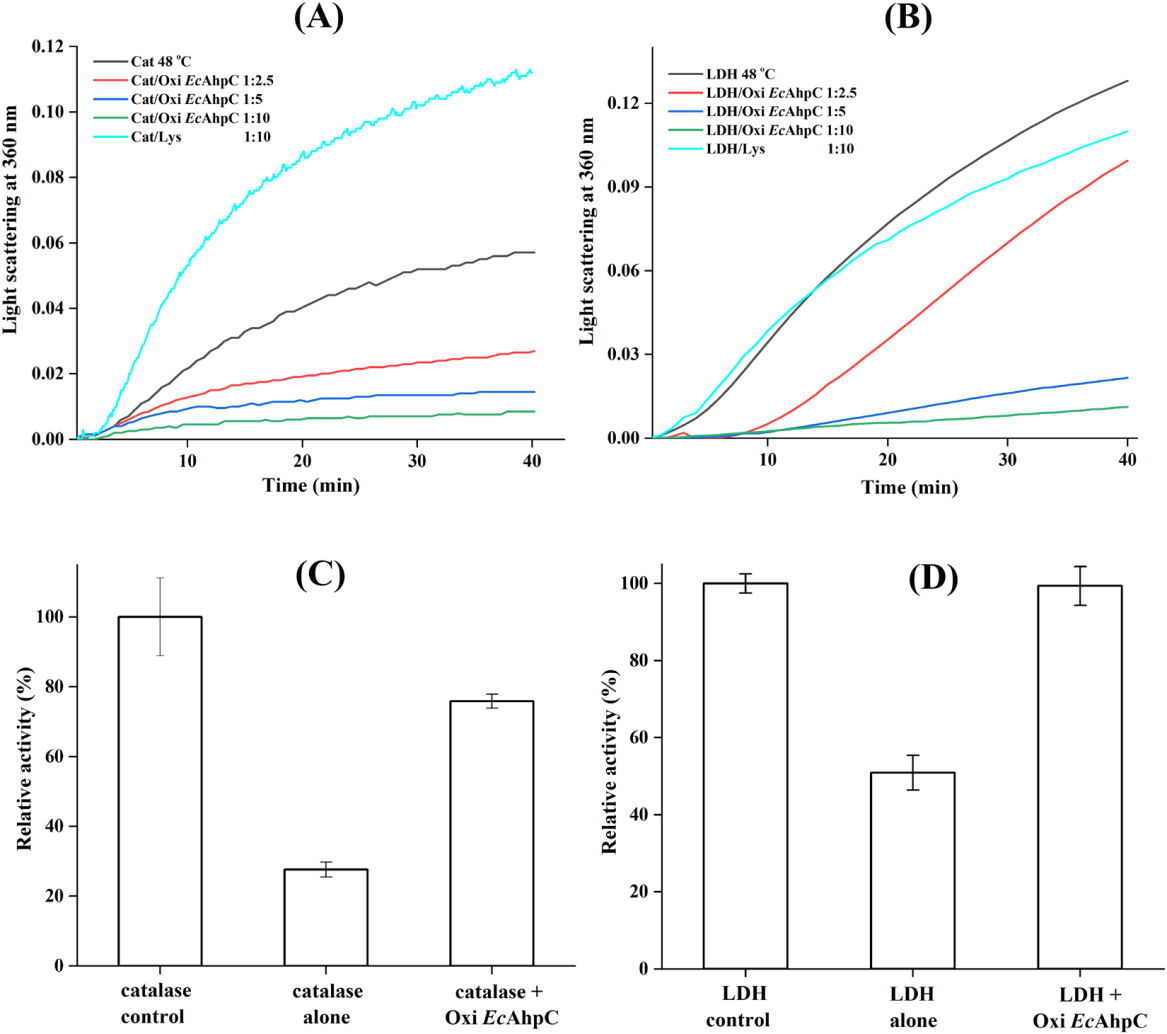
Chaperone-like activity of oxidized *Ec*AhpC. Light scattering assay of thermal induced aggregations of (**A**) 1 µM of catalase (Cat) and (**B**) 1 µM LDH at 48 °C in absence and presence (2.5 to 10 µM) of oxidized *Ec*AhpC. Catalase- and LDH aggregation was prevented in the presence of increasing amounts of oxidized *Ec*AhpC. The different catalase:*Ec*AhpC and LDH:*Ec*AhpC molar ratios are indicated in the figures. Incubation with 10-fold excess of lysozyme (Lys) conferred no protection for catalase or LDH at 48 °C. (**C**) The relative activity was calculated based on H_2_O_2_-decomposition by the catalase at 25 °C, which was considered to be 100%. The catalase activity considerably decreased after heat shock at 48 °C. Incubation along with oxidized *Ec*AhpC prevented the reduction of catalase activity at 48 °C. (**D**) The relative activity was calculated based on pyruvate converted to lactate by LDH resulting in the oxidation of NADH at 25 °C, which was considered to be 100%. The LDH activity decreased by 50% after heat shock at 48 °C. Incubation along with oxidized *Ec*AhpC prevented the reduction of LDH activity at 48 °C. All the data shown are the means of at least three independent experiments.

*Ec*AhpC is a member of the AhpC/Prx1 subfamily of antioxidant defense enzymes, catalysing the rapid reduction of ROS such as H_2_O_2_, organic hydroperoxide, and peroxynitrite^6,25,26^. Besides their antioxidant role, sensitive Prx1 subfamily enzymes have been widely reported to act as a chaperone, preventing protein aggregation^14^. Comparatively, bacterial AhpCs were less exposed for their functional role under heat stress conditions. To demonstrate the chaperone-like activity of *Ec*AhpC *in vitro*, the purified His-tagged recombinant *Ec*AhpC was analysed for its ability to prevent the thermal induced aggregations of catalase and LDH using light scattering. As shown in Fig. 1A,B, the catalase and LDH slowly aggregated over time at 48 °C as indicated by increasing light scattering at 360 nm. In the presence of different monomer molar ratios of oxidized *Ec*AhpC (zero thiol/monomer was measured by the DTNB assay shown in Supplementary Fig. S1), the absorbance in light scattering reduced significantly, suggesting that the thermal induced aggregation of catalase and LDH were suppressed by oxidized *Ec*AhpC in a concentration-dependent manner. In general, with increasing monomer molar concentrations of oxidized *Ec*AhpC aggregation of client proteins (CP) was significantly reduced. In comparison, a 10-fold excess of lysozyme (control) did not supress the aggregation of CP.

Besides the prevention of aggregation, the ability of *Ec*AhpC to protect the biological activity of catalase under heat stress has been verified at 48 °C. The absence of any external reducing equivalent the oxidized *Ec*AhpC does not have any specific activity with its substrate H_2_O_2_ (Supplementary Fig. S2A). As a consequence, any decrease in absorbance at 240 nm at which H_2_O_2_ reduction can be recorded would be the result of H_2_O_2_-decomposition by the catalase (Supplementary Fig. S2A). As shown in Fig. 1C, the catalase lost about 70% of enzyme activity upon incubation at 48 °C for 1 h. In comparison, incubation of the catalase along with oxidized *Ec*AhpC at 48 °C restored the catalase activity to 75% (Fig. 1C). Similarly, the protection of LDH activity by oxidized *Ec*AhpC has been tested as described in materials and methods. As Fig. 1D demonstrates, LDH incubated at 48 °C for 1 h lost 50% of its enzyme activity. Incubation of LDH along with oxidized *Ec*AhpC at 48 °C completely restored the enzyme activity of LDH (Fig. 1D and Supplementary Fig. S2B). These results demonstrated that *Ec*AhpC is able to protect the CP from thermal induced aggregation and loss in enzymatic activity in a manner similar to other reported small heat shock proteins (sHSP)^27–30^. It has been suggested that sHSP capture unfolded CP to reactivate them at an early stage during stress-induced unfolding, and to protect them from aggregation at a later stage^27,28^. However, the proposed mechanism(s) of *Ec*AhpC chaperone activities, ranging from protection of inactivation and suppression to aggregation, remain(s) to be elucidated.

### Heat induced oligomerization of oxidized *Ec*AhpC

**Figure 2:**
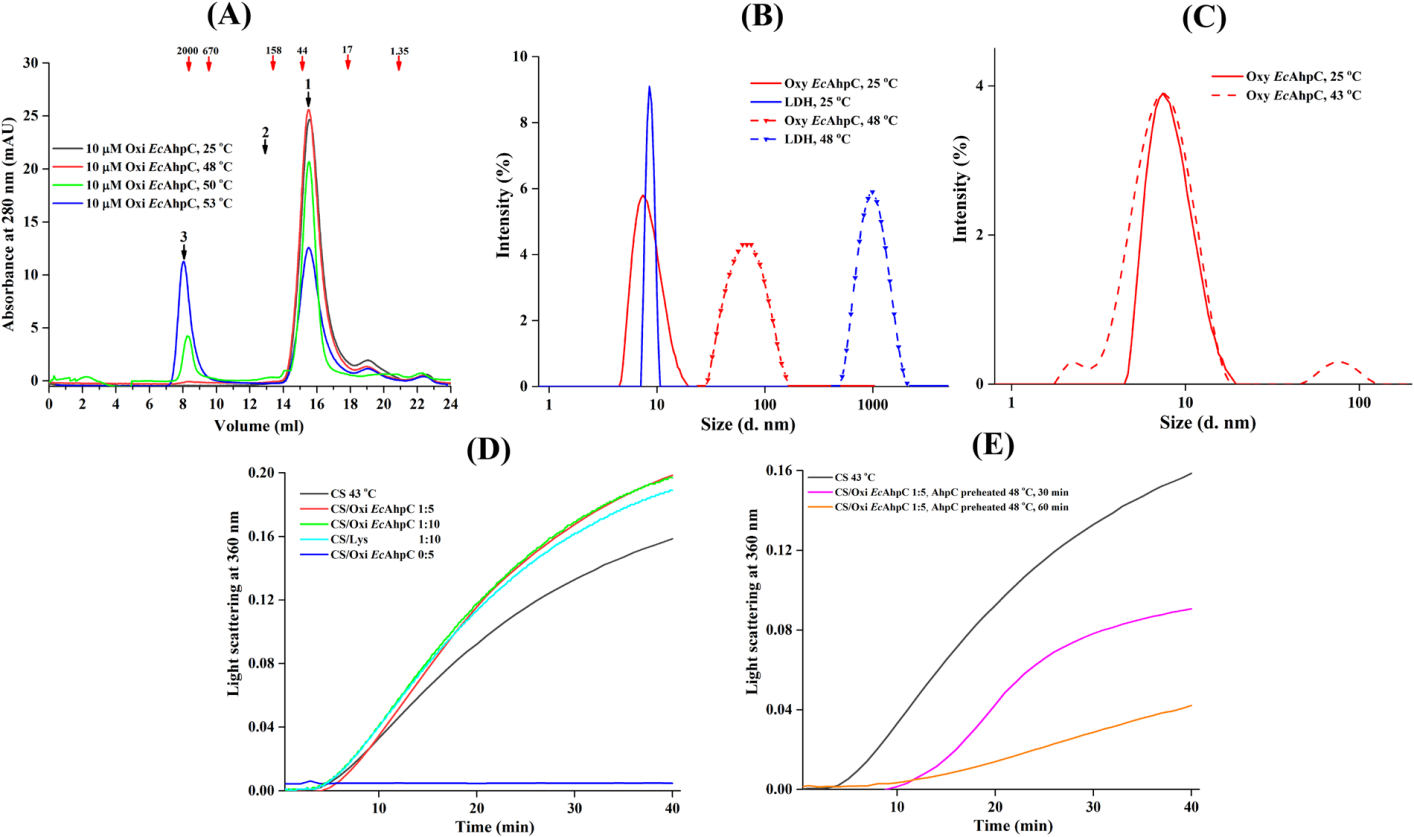
Heat-shock induced oligomerization of oxidized *Ec*AhpC. (**A**) SEC elution profile of 10 µM oxidized *Ec*AhpC incubated at 25 °C (*black*), 48 °C (*red*), 50 °C (*green*) and 53 °C (*blue*). The dimeric (estimated molecular mass of 38 kDa) and decameric (estimated molecular mass of 195 kDa) forms of oxidized *Ec*AhpC elution volumes were denoted as 1 and 2, respectively. A very large shift in the elution volume (denoted as 3) observed for the sample treated with heat indicates HMW oligomers formation with an estimated molecular mass of 2.0–3.0 MDa. The numbers indicated with red arrows in the chromatogram represent the molecular mass (kDa) of standard proteins. (**B**) DLS measurement of oxidized *Ec*AhpC (*red*) and LDH (*blue*) were equilibrated at 25 °C (—) and 48 °C (−) for 10 min, respectively. An increase in mean effective diameter were observed for both the samples at 48 °C for 10 min. (**C**) DLS studies showed no significant changes in the oligomerization of oxidized *Ec*AhpC (*red*) after treatment at 43 °C for 10 min (−). (**D**) Light scattering analysis showed that 0.5 µM of CS aggregated at 43 °C (*black*) and that the aggregation was not prevented in the presence of 5 µM (red) and 10 µM (*green*) of oxidized *Ec*AhpC or 10 µM lysozyme (*cyan*). (**E**) 0.5 µM CS aggregation at 43 °C was prevented in the presence of 5 µM oxidized *Ec*AhpC, which was pre-heated at 48 °C for 30 (*magenta*) and 60 (*orange*) min, respectively.

Formation of large homo-oligomers has been reported to be essential for chaperone function of many sHSP^31^. This hold true for the chaperone activity of Prx1 subfamily enzymes, which form the HMW oligomers under stress conditions^14^. As shown in the elution profile of the size exclusion chromatography (SEC) column (Fig. 2A), *Ec*AhpC eluted at around 15.5 ml at a temperature of 25 °C with an estimated molecular mass of about 38 kDa (Supplementary Fig. S3A,B), which corresponds to a dimer. Interestingly, *Ec*AhpC, incubated at 48 °C, 50 °C and 53 °C for 60 min, eluted at around 8.0 ml and 15.5 ml with a peak ratio of 0.02:1, 0.2:1, 0.9:1, respectively. Considering that the oxidized *Ec*AhpC in a high salt buffer (50 mM HEPES pH 7.0 and 320 mM of ammonium sulfate) condition eluted at 13 ml at 25 °C using the same SEC column^32^ with a calculated molecular mass of about 195 kDa (Supplementary Fig. S3B), the data indicate that at 53 °C the oxidized *Ec*AhpC eluted in peak 3 formed an HMW oligomer with a molecular mass of about 2.0–3.0 MDa, corresponding to 100–150 subunits (Fig. 2A).

In the complementary approach of Dynamic light scattering (DLS) experiments a diameter of 8.5 ± 3.7 nm and 71.9 ± 26.8 nm could be calculated for oxidized *Ec*AhpC at 25 °C and 48 °C, respectively. Considering that the decameric *Ec*AhpC has a diameter of 11.3 ± 4.9 nm, which correlates to a molecular weight of around 193 ± 85 kDa^8,25^, these results confirm the temperature effect in HMW oligomer assembling of *Ec*AhpC as seen in the SEC experiments and add the information, that the observed 0.02:1, 0.2:1, 0.9:1 peak ratios at 8 ml and 15.5 ml for *Ec*AhpC at 48 °C, 50 °C and 53 °C, respectively (Fig. 2A) may be caused by the fact that the enzyme was pre-heated but eluted on a column at room temperature, allowing the protein to reassemble into a dimer. However, the HMW oligomers formed with an increasing temperature remain relatively stable even after cooling to room temperature (Fig. 2A).

At the same time, the DLS data showed a mean effective diameter of 1,034 ± 294 nm for LDH at 48 °C, demonstrating that this enzyme forms larger aggregates at this temperature (Fig. 2B). These findings suggested that the low oligomeric forms of oxidized *Ec*AhpC have the tendency to assemble into HMW oligomers under heat stress condition.

### Temperature-dependent oligomerization and chaperone activity of *Ec*AhpC

Temperature-dependent oligomerization and chaperone activity of *Ec*AhpC were verified using DLS and the chaperone activity assay at 43 °C. Incubation of oxidized *Ec*AhpC at 43 °C for 10 min did not alter the oligomeric form, which predominantly existed as a lower order oligomer (8.4 ± 3.8 nm), and which is similar to that of the oxidized *Ec*AhpC at 25 °C (Fig. 2C). In addition, the chaperone-like activity assay demonstrated that the citrate synthase aggregated slowly over time at 43 °C as shown by the increased light scattering at 360 nm (Fig. 2D). Incubation of the CS with an excessive amount of oxidized *Ec*AhpC did not alter the light scattering at 360 nm, indicating that the oxidized *Ec*AhpC did not prevented the heat induced aggregation of the CS at 43 °C (Fig. 2D). However, incubation of CS with oxidized *Ec*AhpC pre-heated at 48 °C for 30 and 60 min, respectively, prevented CS aggregation with increased chaperone-like activity for a prolonged incubated sample (Fig. 2E). These experiments demonstrated that the HMW oligomer formation and the chaperone-like activity of oxidized *Ec*AhpC depend on the incubation temperature and the period of incubation.

### HMW oligomer binds the client protein under thermal stress

**Figure 3:**
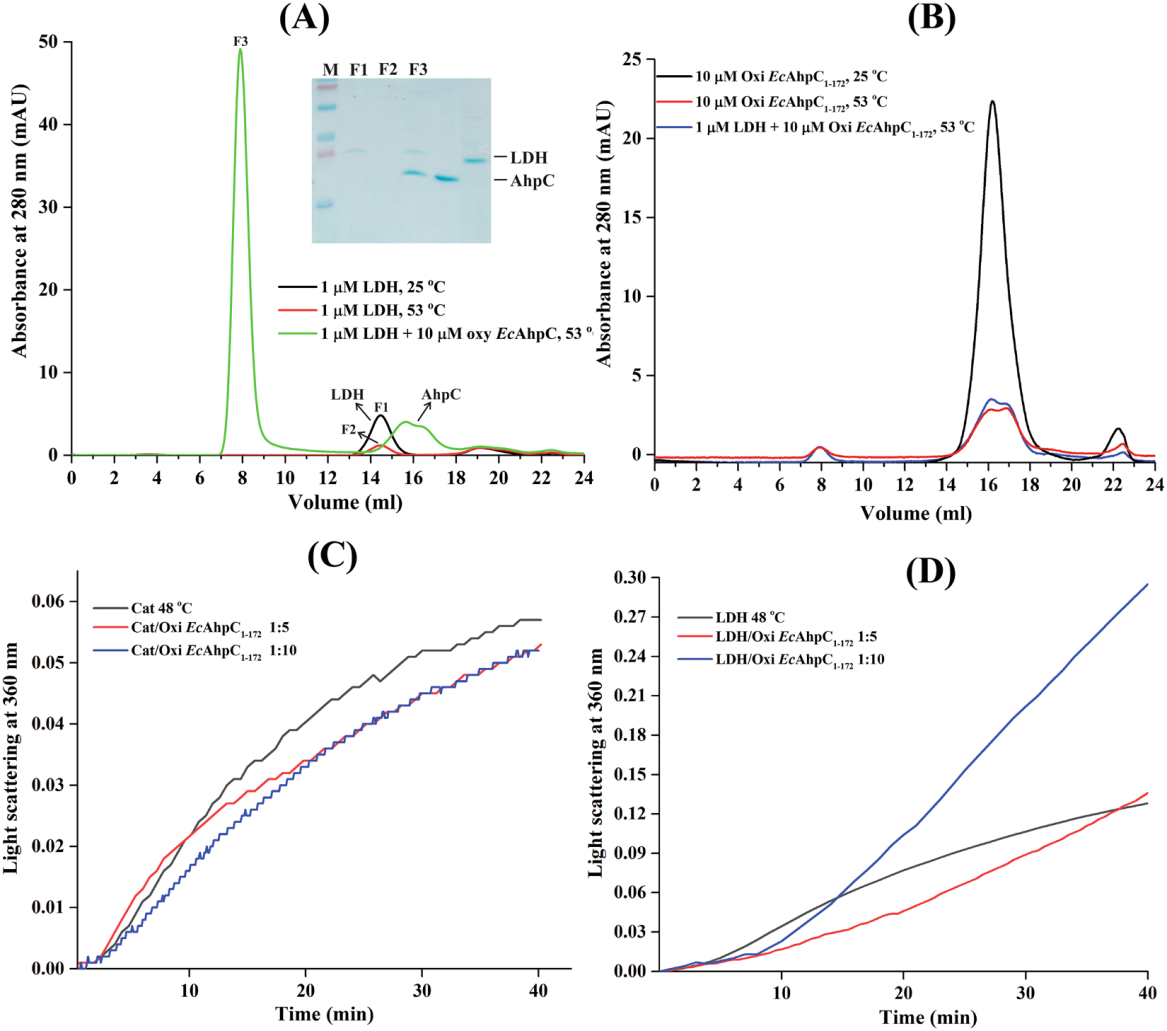
Role of the C-terminal tail on heat induced oligomerization. (**A**) SEC analysis showed that 1 µM of LDH largely aggregated and that only a small amount remained soluble after heat treatment at 53 °C for 1 h (*red*) compared to that of at 25 °C (*black*). In comparison, SEC and SDS-PAGE (*inset*; full-length gel is presented) analysis revealed that the denatured LDH remained completely soluble after treatment at 53 °C for 1 h with HMW oligomers of oxidized *Ec*AhpC. (**B**) The C-terminal tail deletion mutant, *Ec*AhpC_1–172_, was largely precipitated after heat treatment at 53 °C in absence (*red*) and presence (*blue*) of LDH. The light scattering experiment showed that oxidized *Ec*AhpC_1–172_ did not prevent the heat induced aggregation of (**C**) 1 µM catalase and (**D**) 1 µM LDH at 48 °C.

To further explore the role of HMW oligomers in a chaperone activity, we incubated 1 µM of LDH with or without 10 µM of oxidized *Ec*AhpC at 53 °C for 60 min, centrifuged the sample and subjected the supernatant to a SEC column at room temperature. The absorbance drop of the LDH-peak at 14.5 ml in Fig. 3A of the preheated protein at 53 °C compared to the one of the non-preheated LDH revealed, that the enzyme was largely precipitated at 53 °C before injection into the column. In comparison, when LDH and oxidized *Ec*AhpC were incubated together at 53 °C, centrifuged and its supernatant applied on the SEC column, one major peak eluted at 8 ml (Fig. 3A), which corresponds to the HMW oligomeric form of *Ec*AhpC (Fig. 2A), and a small peak which eluted at the volume of dimeric *Ec*AhpC (Fig. 3A). The subsequent SDS-PAGE analysis of the eluted peak at 8 ml revealed that the soluble LDH co-eluted with the HMW *Ec*AhpC after heat stress. This suggested that the HMW oligomers of *Ec*AhpC bound LDH and might prevent their irreversible aggregation under thermal stress conditions.

### The C-terminal tail of *Ec*AhpC is necessary for chaperone activity

In previous studies^32,33^, we have observed that the flexible C-terminal extension of *Ec*AhpC (aa 173–187) is critical for decamer formation of the reduced form and the ensemble formation with its redox-partner *Ec*AhpF of the oxidized form. Here, we hypothesized that the C-terminal tail might also have a role in chaperone function. To test this, the C-terminal truncated protein *Ec*AhpC_1–172_ was analysed for HMW oligomerization and chaperone activity as described above for the entire enzyme. The respective elution profile of the SEC analysis at 25 °C revealed that *Ec*AhpC_1–172_ eluted at around 16.1 ml, which corresponds to a molecular mass of 28 kDa. However, the heat treated *Ec*AhpC_1–172_ alone or with LDH at 53 °C for 60 min showed that *Ec*AhpC_1–172_ neither formed HMW oligomers nor a complex with LDH. The low absorbance at 16.1 ml demonstrated also, that the protein largely precipitated already at 53 °C before injection into the column (Fig. 3B).

The data of a possible chaperone-like activity of *Ec*AhpC_1–172_ in Fig. 3C,D showed that the presence of excess of this protein did not significantly decrease the heat induced aggregation of LDH and catalase at 48 °C. These results demonstrated that the C-terminal tail of *Ec*AhpC is essential for the formation of HMW oligomer species, which exhibited molecular chaperone function.

### Redox state independent chaperone function

**Figure 4:**
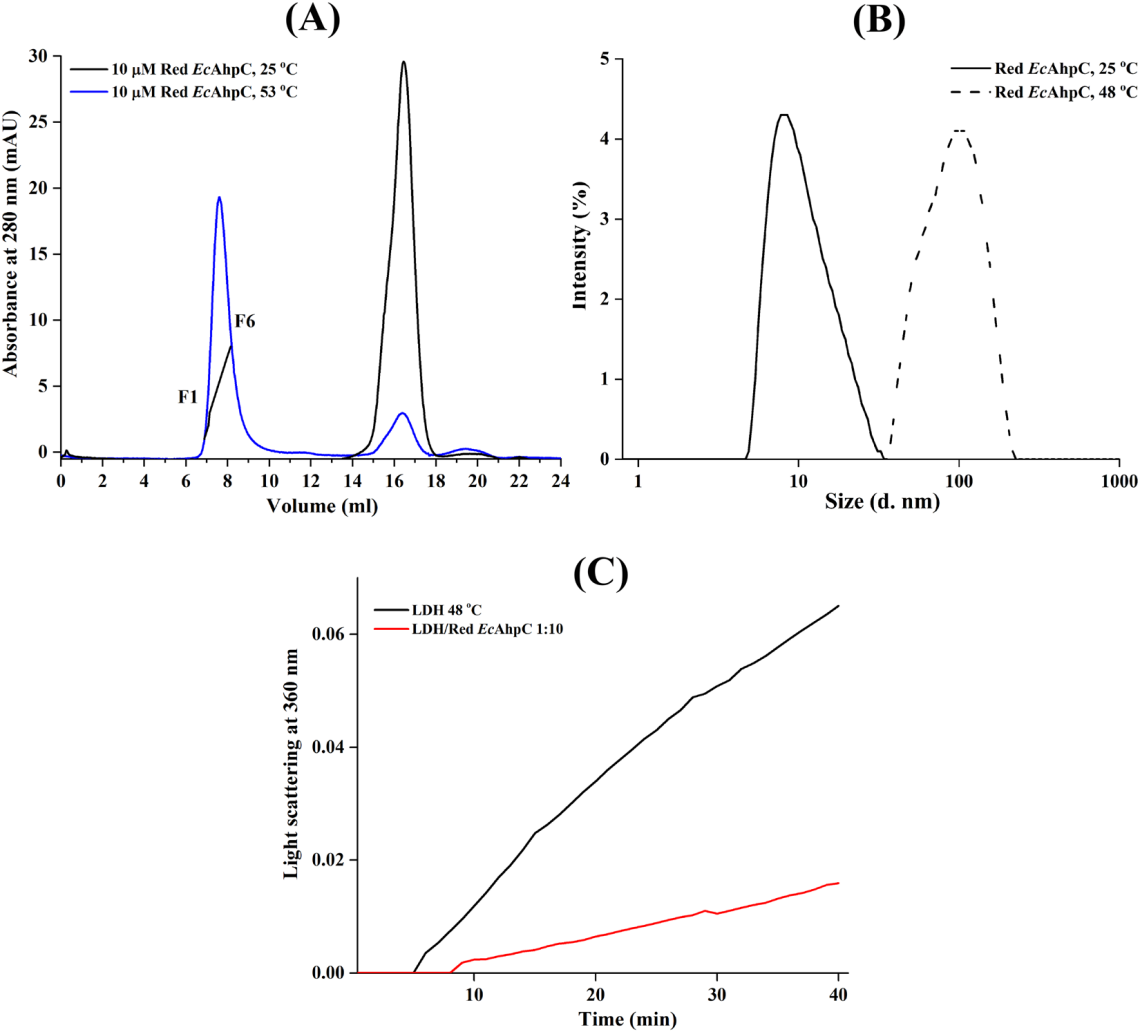
Chaperoning activity of reduced *Ec*AhpC. (**A**) SEC elution profile of 10 µM of reduced *Ec*AhpC treated at 25 °C (*black*) and 53 °C (*blue*) for 1 h. A very large shift in the elution volume is observed for sample treated at 53 °C (*blue*), demonstrating the formation of HMW oligomers. The trace indicated on the elution peak was collected (F1 to F6) for SDS-PAGE analysis. (**B**) DLS data present the increase in diameter of reduced *Ec*AhpC after heating of the enzyme at 48 °C for 10 min (−). (**C**) 1 µM LDH aggregation (*black*) was greatly reduced in the presence of reduced *Ec*AhpC at 48 °C (*red*).

The redox state of AhpC is a key factor determining the dimer–decamer equilibrium. The reduced form of WT *Ec*AhpC (two thiols/monomer as measured by the DTNB assay shown in Supplementary Fig. S1) eluted at around 16.5 ml in SEC at a temperature of 25 °C with an estimated molecular mass of 22 kDa (Supplementary Fig. S3). However, the DLS studies revealed a diameter of 11.3 ± 4.9 nm that corresponds to a molecular weight of around 193 ± 85 kDa of the reduced *Ec*AhpC at 25 °C (Fig. 4B). This result indicated that *Ec*AhpC at 25 °C exists predominantly as a decamer as confirmed by 2D projections from electron micrographs of the enzyme^8^. To test the ability of reduced *Ec*AhpC to function as a chaperone under heat stress, *Ec*AhpC was incubated at 53 °C for 60 min in the presence of 1 mM of the reducing agent TCEP and applied to SEC after centrifugation. The respective elution profile in Fig. 4A represents a major peak at 8 ml with an estimated molecular mass of 2.0 to 3.0 MDa and a minor one at around 16.5 ml both in a ratio of about 10:1, demonstrating that the increase in temperature resulted in HMW forms of reduced *Ec*AhpC. When compared to the profile of oxidized *Ec*AhpC, which was pre-heated at 53 °C for 60 min (Fig. 2A), the amount of formed higher oligomers of reduced *Ec*AhpC at this temperature was higher (Supplementary Fig. S4). The DLS data at 48 °C (Fig. 4B), at which also the chaperone assay was performed (Fig. 4C), confirmed the temperature-induced formation of higher oligomers by an increase in size of reduced *Ec*AhpC at 25 °C with a diameter of 11.3 ± 4.9 nm, indicating decamer formation, and a diameter of 103 ± 37 nm for the reduced enzyme at 53 °C, confirming higher oligomer formation (Fig. 4B). In general, the data suggested that a redox-state independent HMW oligomer formation can be observed with quantitative increase of HMW oligomers of reduced *Ec*AhpC when compared to the oxidized enzyme, which might arise from the difference observed between the thermal stability of the oxidized and reduced form of *Ec*AhpC^25^. Furthermore, the chaperone like activity assay revealed that the presence of 10 µM of reduced *Ec*AhpC can decrease the heat induced aggregation of LDH at 48 °C (Fig. 4C).

## Discussion

### Proposed physiological role of bacterial AhpC under stress conditions

In response to H_2_O_2_, the peroxide sensing transcriptional regulator, OxyR, activates the expression of several antioxidants such as alkylhydroperoxide reductase (AhpCF) and catalase (KatG)^3^. The peroxide reduction activity of the robust 2-Cys Prx, namely *Ec*AhpC, has been well established under the oxidative stress conditions^34,35^. The *Ec*AhpC assumes the primary scavenger role by consuming the endogenous levels of H_2_O_2_ produced due to the aerobic metabolism^34^. *Ec*AhpC transfers the oxidizing equivalent from H_2_O_2_ to NADH through *Ec*AhpF, an FAD-based NADH oxidase that catalyse the rapid-reduction of the disulfide bond of *Ec*AhpC formed during peroxide reduction^36,37^. The peroxidase activity of *Ec*AhpC is expected to saturate at high levels of H_2_O_2_ due to the dependence on the energy currency NADH for its enzymatic recycling^3,34,38^. Under such H_2_O_2_ stress conditions, the catalase has been suggested to function as a primary scavenger, indicating that there could be a complementing role for antioxidant enzymes during stress resistance^34,38^. Moreover, many studies indicated that the tolerance to severe heat shock is tightly linked to aerobic metabolism and oxidative stress^39^. This suggests that heat shock induces a subsequent oxidative stress and that many antioxidant enzymes play an essential role in heat induced oxidative stress resistance^15,40^. In *E. coli*, the enzymes involved in oxidative stress defense were highly up-regulated including *Ec*AhpC (17.7 fold) under the elevated temperature of 47.5 °C^41^ and enhanced survival under thermal stress^42^.

### The proposed mechanism of chaperone activity of bacterial Prxs

**Figure 5:**
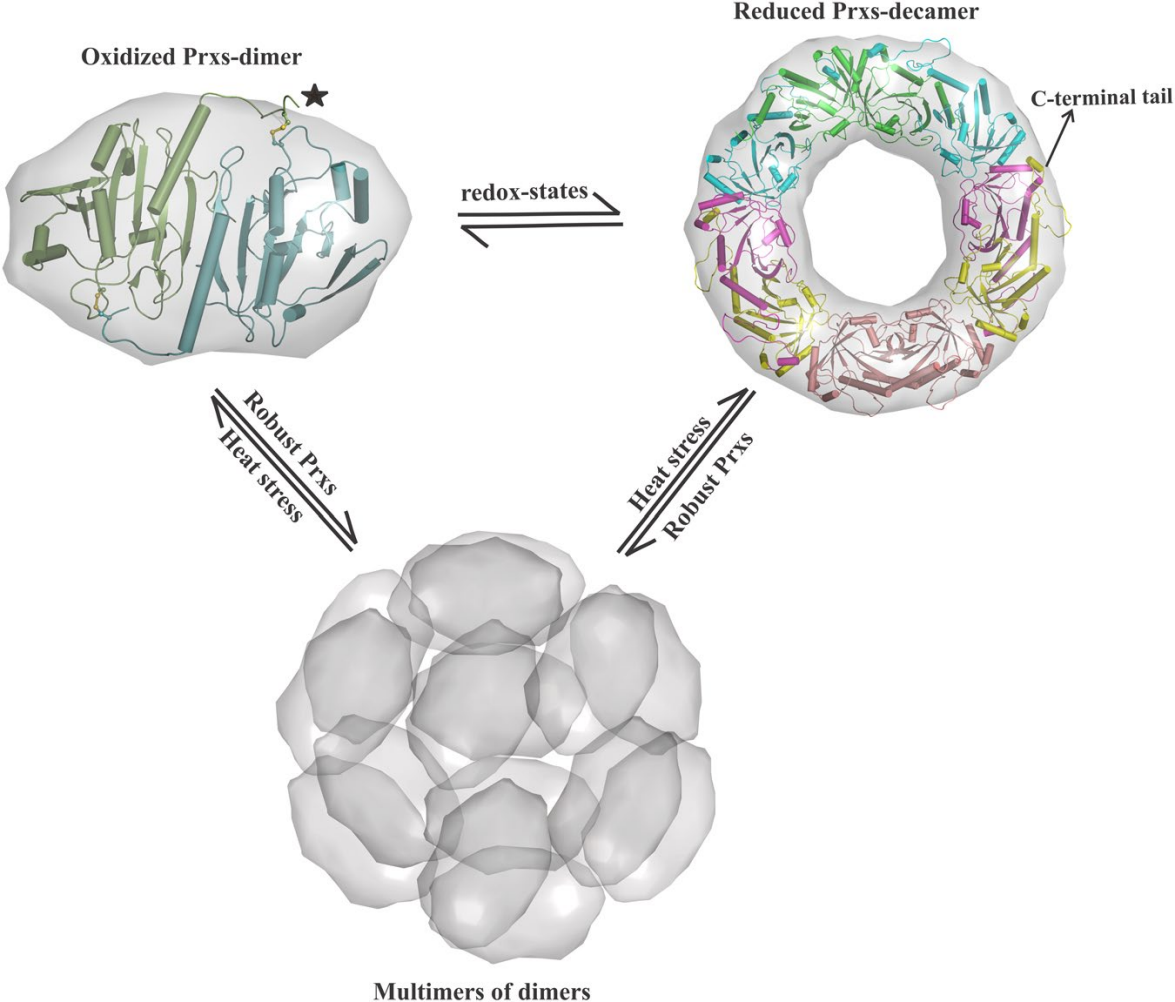
Proposed chaperoning mechanism of AhpC/Prx1 enzymes. AhpC/Prx1 subfamily enzymes undergo redox-state dependent oligomerisation during their catalytic cycle. The decamer formed in the reduced state destabilises upon the formation of intermolecular disulphide bonds. In their reduced form, the C-terminal tail of Prx1 enzymes is required for their sensitivity to over-oxidation. The robust bacterial Prxs provide chaperon activity in the oxidized and reduced form, highlighting that the dimeric form can assemble into HMW oligomers under heat stress condition. The C-terminal tail of these enzymes is highly disordered in the oxidized structure, but essential for the heat induced HMW oligomer formation.

An alternate chaperone function has been established for sensitive Prx1 subfamily enzymes^15–24^. In general, their chaperone activity depends on the redox-state of the enzyme, the reduced C_P_ is susceptible to over-oxidation (C_P_-SO_2_H/SO_3_H) under severe oxidative and heat stress conditions, which eventually promotes the stacking of decamers into HMW oligomers^43^. However, little is known about the chaperone activation mechanism of robust bacterial AhpCs, which are highly resistant to inactivation by H_2_O_2_^7,24^. In the present study, we decipher the chaperone function of *Ec*AhpC in heat-shock conditions, which normally promotes the irreversible aggregations of proteins. We show that *Ec*AhpC forms HMW oligomers under heat stress and prevents the heat induced aggregation and inactivation of client proteins (Figs 1 and 2). Moreover, the formation of HMW oligomers is independent of the redox-state, indicating that the dimeric form of *Ec*AhpC might serve as the basic structural unit of HMW chaperone species (Fig. 5). This implies that both the oxidized and reduced forms of *Ec*AhpC undergo structural alteration at heat-shock temperature to adopt HMW oligomers that look similar in size in SEC and DLS studies (Figs 2A and 4A).

In addition, for the first time we revealed the role of the C-terminal tail of *Ec*AhpC on heat induced structural alterations to form HMW oligomers. There is an evolutionary variation of the role of the C-terminal tail of Prxs to activate the chaperone function for both the robust and sensitive Prxs. In sensitive Prxs, the extended C-terminal helix with the conserved YF-motif folds across their active site region and is essential for the over-oxidation, but the C-terminal tail is highly disordered in the oxidized state^7,24,25^. In bacterial AhpC, the C-terminal tail is essential to generate heat-induced HMW oligomer formation, suggesting that changes in temperature-induced modification at the surface of *Ec*AhpC that favour formation of HMW oligomers and chaperone-like activity. This suggests a significant difference in the mode of action of bacterial AhpCs compared to sensitive Prxs. In the case of sensitive Prxs, high levels of H_2_O_2_ produced during heat stress conditions enable the over-oxidation of their reduced form and the decamer acts as the basic structural unit to form HMW oligomers. However, in the case of AhpC, we presume that the high level of H_2_O_2_ production and the lack of continues supply of NADH during severe heat stress conditions saturate the antioxidant activity of AhpC^34^, which, as a result, might predominantly exist in an oxidized dimeric form. Therefore, it is not excluded that, in the case of AhpC, the oxidized and reduced form might serve as the basic structural unit of HMW oligomers under heat stress conditions (Fig. 5). Importantly, we have shown that the catalase, the primary antioxidant proposed to work under high H_2_O_2_ stress, is prone to heat induced aggregation that has been rescued by chaperone activity of AhpC.

## Materials and Methods

### Cloning, expression and purification

Recombinant His-tagged wild-type (WT) *Ec*AhpC and the C-terminal truncated *Ec*AhpC_1–172_ were produced and purified by the method described earlier^7,8,11^. Briefly, the *E. coli* cells producing the respective recombinant proteins were lysed on ice by sonication with an ultrasonic homogenizer (Bandelin, KE76 tip) for 3 × 1 min in buffer A (50 mM Tris–HCl pH 7.5, 200 mM NaCl, 2 mM Phenylmethylsulfonyl fluoride (PMSF), 1 mM Pefabloc^SC^, 0.8 mM DTT). After sonication, the cell lysate was centrifuged at 10,000 × g for 35 min at 277 K. The resulting supernatant was passed through a filter (0.45 μm; Millipore) and supplemented with Ni^2+^-NTA resin pre-equilibrated in buffer A. The His-tagged proteins were allowed to bind to the matrix for 1 h at 277 K by mixing on a sample rotator (Neolab). To avoid remaining DTT from the lysis buffer A, the Ni^2+^-NTA was initially washed with 10 column volumes of buffer A without DTT and subsequently eluted with an imidazole gradient (0–500 mM). Fractions containing the required proteins were further purified using gel filtration chromatography using a Superdex 75 HR 10/30 column (GE Healthcare) with a buffer consisting of 50 mM Tris–HCl pH 7.5, 200 mM NaCl. The purified disulfide bonded (referred as oxidized form) of His-tagged recombinant WT *Ec*AhpC and *Ec*AhpC_1–172_ were used for further studies. As recently described^25^, the His-tag does not affect structural or mechanistic traits of *Ec*AhpC or *Ec*AhpC_1–172_.

### Thiol Content Titration

The free thiol content of recombinant WT *Ec*AhpC was estimated using 5,5′ -dithiobis(2-nitrobenzoic acid) (DTNB). The oxidized and reduced form of WT *Ec*AhpC (10 µM of monomer) in 500 µl of phosphate (pH 8.0) buffer was reacted with 100 µM DTNB, followed by detection of the TNB produced at λ = 412 nm. Standard curve generated using cysteine was used to calculate the thiol content of the protein. For the reduced *Ec*AhpC, oxidized WT *Ec*AhpC was reduced with 5 mM DTT in 50 mM phosphate (pH 8.0) buffer for 30 min. Reduced proteins were separated from excess of DTT using a PD-10 desalting column (GE Healthcare).

### *In-vitro* chaperone activity assay of WT *Ec*AhpC and *Ec*AhpC_1–172_

Thermal induced aggregation of 0.5 µM citrate synthase (Sigma-Aldrich), 1 µM catalase (Sigma-Aldrich) and 1 µM lactate dehydrogenase (Calbiochem) in 50 mM phosphate (pH 7.0) buffer, was monitored in the absence or presence of different molar ratios of oxidized *Ec*AhpC, *Ec*AhpC_1–172_ or lysozyme. Protein aggregation was measured by light scattering at 360 nm using an UV−vis spectrophotometer with a thermostatic cell holder assembly maintained at 43 °C for CS and 48 °C for catalase and LDH^44^. To prepare reduced *Ec*AhpC, oxidized *Ec*AhpC was incubated in the presence of 5 mM dithiothreitol (DTT) for 30 min. The chaperone activity of reduced *Ec*AhpC was measured by diluting different molar ratios to the final assay mixture in 50 mM phosphate (pH 7.0) buffer containing 250 µM DTT to prevent the re-oxidation.

### Catalase activity assay

Catalase activity was assayed by monitoring the decrease in absorbance at 240 nm resulting from the decomposition of H_2_O_2_ at 25 °C or 48 °C in the presence and absence of oxidized *Ec*AhpC^45^. 10 nM of catalase was mixed with or without 100 nM of *Ec*AhpC in 50 mM phosphate buffer, pH 7 and incubated at 48 °C for 60 min. The solution was cooled to 25 °C and the catalase activity was measured by adding H_2_O_2_ (final concentration of 10 mM). Catalase assays were carried out at 4 min intervals. The activity is expressed relative to the catalase activity measured at 25 °C without incubation at 48 °C in the respective time interval.

### LDH activity assay

LDH activity was measured based on the reaction that the pyruvate kinase converts one molecule of phosphoenolpyruvate to pyruvate when the ADP is converted into ATP. Pyruvate was subsequently converted to lactate by LDH resulting in the oxidation of one NADH molecule. LDH (final concentration of 5 nM), incubated at 48 °C in 500 ml of 50 mM phosphate buffer (pH 7.0) in the presence and absence of 50 nM of oxidized *Ec*AhpC, was cooled to room temperature. 1 mM phosphoenolpyruvate, 1 mM ADP, 200 µM NADH, and 0.2 mg/ml pyruvate kinase were added to measure the rate of NADH-absorbance decrease at λ = 340 nm. The activity was expressed relative to the LDH-activity measured at 25 °C without incubation at 48 °C in the respective time interval.

### Dynamic light scattering

DLS studies of 0.5 mg/ml LDH (=13.7 µM) and the 1 mg/ml of oxidized or reduced form of WT *Ec*AhpC (=50 µM) were performed using 12 µl of respective proteins in 50 mM phosphate (pH 7.0) buffer using a Malvern Zetasizer Nano ZS spectrophotometer equipped with a thermostat cell holder in a low-volume quartz batch cuvette (ZEN2112, Malvern Instruments). The reduced proteins were prepared by adding 5 mM DTT to the respective oxidized forms. After 10 min equilibration at 25 °C or 48 °C, the backscattering at 173° was detected for all proteins. Scattering intensities were analysed using the in-built Zetasizer software to estimate the hydrodynamic diameter, size, and volume distribution.

### Size exclusion chromatography

To determine the oligomerization state of oxidized *Ec*AhpC and *Ec*AhpC_1–172,_ 10 µM of proteins in 50 mM phosphate (pH 7.0) were incubated at 25 °C, 48 °C, 50 °C or 53 °C for 60 min. After cooling to room temperature, the samples were subjected to high speed centrifugation and loaded on a SEC column (Superdex 200 10/300 GL column (GE Healthcare)), equilibrated with 50 mM Tris, pH 7. The samples were detected by absorbance at 280 nm. For the reduced *Ec*AhpC, oxidized WT *Ec*AhpC was reduced with 20 mM DTT in 50 mM phosphate buffer (pH 7.0) for 1 h. Reduced proteins were separated from excess of DTT using a PD-10 desalting column (GE Healthcare). This pre-reduced *Ec*AhpC was incubated at 25 °C or 53 °C for 60 min in a 50 mM phosphate (pH 7.0) containing 1 mM TCEP and subjected to SEC analysis.

Protein molecular mass standards (Bio-rad), thyroglobulin (670 kDa), γ-globulin (158 kDa), ovalbumin (44 kDa), myoglobin (17 kDa), and vitamin B_12_ (1.35 kDa) were used to calibrate the SEC column. The column void volume was determined with blue dextran 2000 (Amersham Pharmacia Biotech Co., Little Chalfont, England).

### *In vitro* binding assay

1 µM of LDH were incubated with or without 10 µM *Ec*AhpC or *Ec*AhpC_1–172_ in 50 mM phosphate (pH 7.0) buffer at 53 °C for 60 min. The samples were analysed by SEC on a Superdex 200 10/300 GL column (GE Healthcare) with a mobile phase of 50 mM Tris (pH 7.0). The elution peak was collected and further analysed by SDS-PAGE^46^.

## Electronic supplementary material

Supplementray Figures
