# Supplementary material for: Molecular mechanism of the Escherichia coli AhpC in the function of a chaperone under heat-shock conditions
Source: Sci Rep. 2018 Sep 20;8:14151. doi: 10.1038/s41598-018-32527-7 (PMC6147784; doi:10.1038/s41598-018-32527-7)
Supplement: Supplementary file 1 — Supplementray Figures [file 41598_2018_32527_MOESM1_ESM.pdf]

Molecular mechanism of the *Escherichia coli* AhpC in the function of a chaperone under heat-shock conditions

Neelagandan Kamariah[a], Birgit Eisenhaber[a], Frank Eisenhaber[a,b] and Gerhard Grüber[a,c,*]

[a] Bioinformatics Institute, Agency for Science, Technology and Research (A*STAR), 30 Biopolis Street, #07-01 Matrix, Singapore 138671, Republic of Singapore

[b] School of Computer Engineering, Nanyang Technological University (NTU), 50 Nanyang Drive, Singapore 637553, Republic of Singapore

[c] School of Biological Sciences, Nanyang Technological University, 60 Nanyang Drive, Singapore 637551, Republic of Singapore

* To whom correspondence may be addressed:

Prof. Dr. Gerhard Grüber, Tel.: + 65 - 6316 2989; Fax: + 65 - 6791 3856

E-mail: ggrueber@ntu.edu.sg

Keywords: Oxidative stress, antioxidant, peroxiredoxins, heat-shock, chaperone, molecular evolution

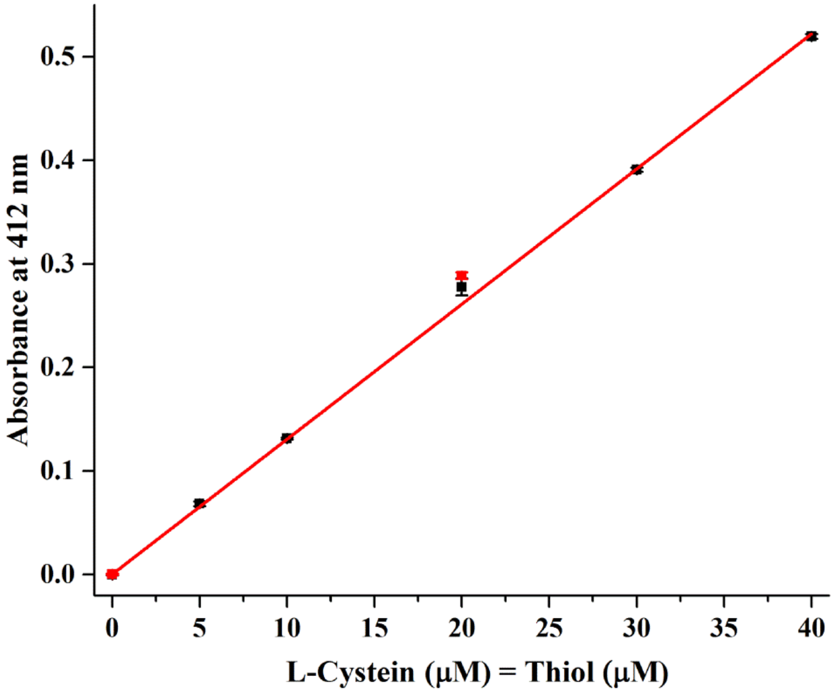

Supplementary Figure S1. *Standard curve for free thiol quantification*. The standard curve is generated by reacting DNTB with different molar concentrations of 0-40 μM L-cysteine (*black square*), followed by detection of the TNB produced at λ = 412 nm, which was used to calculate the free thiol content of 10 μM of recombinant WT *Ec*AhpC (*red square*). The oxidized *Ec*AhpC was estimated to contain 0 ± 0.03 thiol per monomer and the reduced *Ec*AhpC contains 2.1 ± 0.3 thiols per monomer.

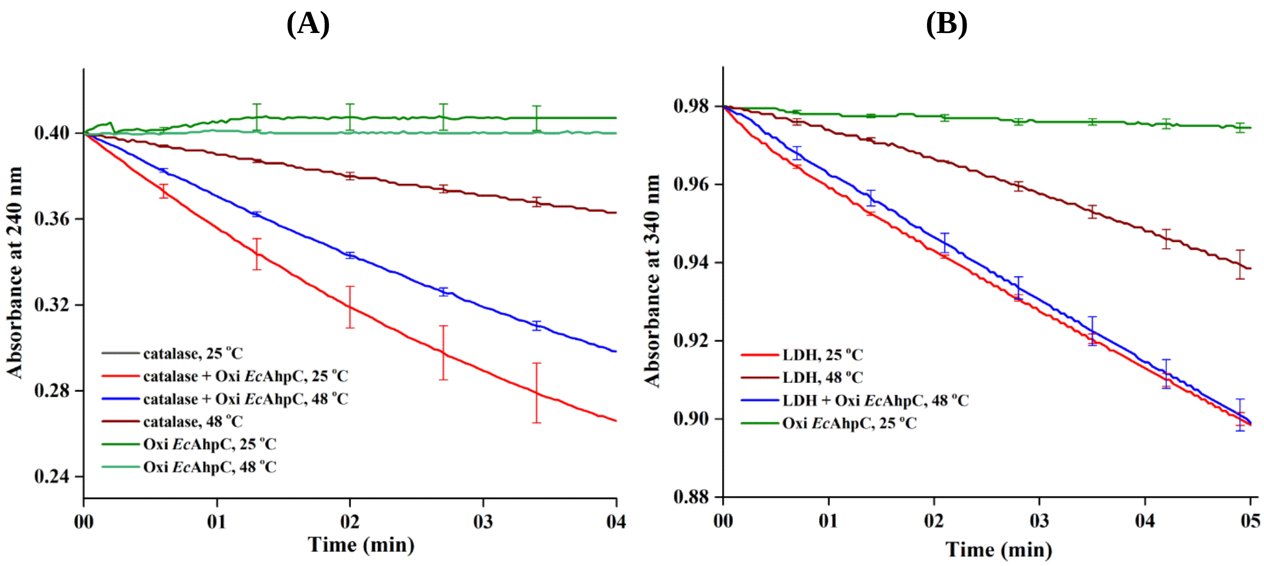

Supplementary Figure S2. *Catalase and LDH activity assay*. (*A*) The decrease in absorbance at $\lambda = 240$ nm resulting from decomposition of H_2O_2 was measured to assess the catalase activity in the presence of oxidized *Ec*AhpC at 25 ºC and 48 ºC. Oxidized *Ec*AhpC alone did not show H_2O_2-decomposition activity at 25 ºC and 48 ºC (*green*), since no decrease in absorbance was observed. (*B*) The decrease in NADH absorbance at $\lambda = 340$ nm was measured to assess the enzymatic activity of LDH in the presence of oxidized *Ec*AhpC at 25 ºC and 48 ºC. The absence of LDH did not show NADH oxidase activity at 25 ºC (*green*), since no decrease in absorbance was observed at 340 nm.

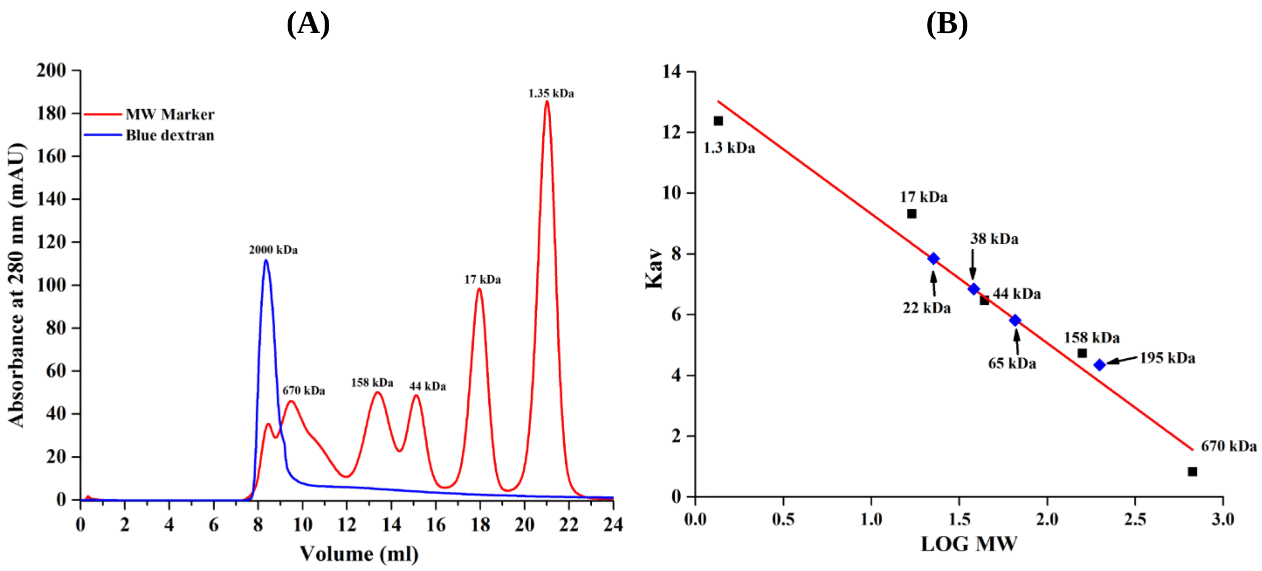

Supplementary Figure S3. *Calibration curve of the SEC.* (*A*) Elution profiles from Superdex 200 10/300 GL column (GE Healthcare) for the calibration of standards (thyroglobulin 670 kDa, γ-globulin 158 kDa, ovalbumin 44 kDa, myoglobin 17 kDa and vitamin B_{12} 1.35 kDa) and blue dextran 2000 as absorption in milliabsorption units (mAU) at λ = 280 nm versus volume in milliliters (ml). (*B*) The calibration curve of the Superdex 200 10/300 GL column was prepared using protein standards (*black squares*). The Kav value was calculated using the equation Kav = (Ve-Vo)/(Vc-Vo), where Vo = column void volume = 8.3 ml, Vc = geometric column volume = 23.6 ml and Ve = elution volume for each protein: thyroglobulin (670 kDa) Ve = 9.48 ml, γ-globulin (158 kDa) Ve = 13.38, ovalbumin (44 kDa) Ve = 15.12, myoglobin (17 kDa) Ve = 17.97, and vitamin B_{12} (1.35 kDa) Ve = 21.03. For each protein samples, the observed Ve was used to calculate the corresponding Kav value that was used to determine the molecular mass. The estimated molecular mass of oxidized *Ec*AhpC decamer (195 kDa), LDH (65 kDa), oxidized *Ec*AhpC dimer (38 kDa) and reduced *Ec*AhpC monomer (22 kDa) is shown (*blue diamond*).

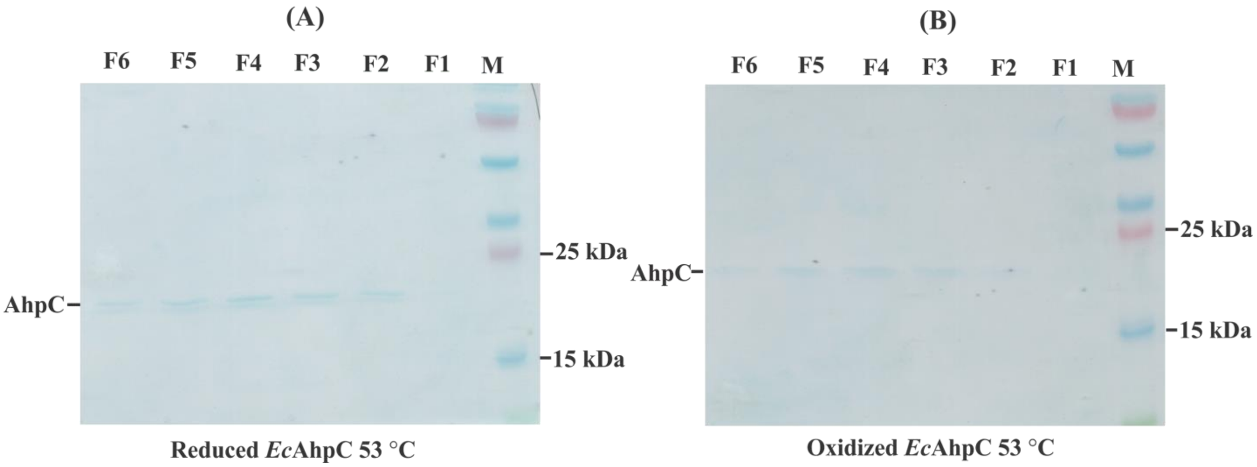

Supplementary Figure S4. *HMW oligomers forming ability of reduced and oxidized EcAhpC at 53 °C.* (A) SDS-PAGE analysis (full-length gel is presented) of the 8 ml elution fractions (F1 to F6) as indicated in the chromatogram (Fig. 4A) of reduced *Ec*AhpC incubated at 53 °C for 60 min. (B) A similar 8 ml elution fractions (F1 to F6) of oxidized *Ec*AhpC incubated at 53 °C for 60 min has been collected and tested on SDS-PAGE analysis. The amount of formed HMW oligomers of reduced *Ec*AhpC at this temperature was higher compared to oxidized *Ec*AhpC.
